# Neuron-Specific Deletion of *Scrib* in Mice Leads to Neuroanatomical and Locomotor Deficits

**DOI:** 10.3389/fgene.2022.872700

**Published:** 2022-05-25

**Authors:** Jerome Ezan, Maité M. Moreau, Tamrat M. Mamo, Miki Shimbo, Maureen Decroo, Nathalie Sans, Mireille Montcouquiol

**Affiliations:** ^1^ INSERM U1215, Neurocentre Magendie, Bordeaux, France; ^2^ University of Bordeaux, Neurocentre Magendie, INSERM U1215, F-33000, Bordeaux, France

**Keywords:** *Scrib*, verheij/8q24.3 deletion syndrome, planar cell polarity, neurodevelopmental disorders, corpus callosum

## Abstract

Scribble (Scrib) is a conserved polarity protein acting as a scaffold involved in multiple cellular and developmental processes. Recent evidence from our group indicates that *Scrib* is also essential for brain development as early global deletion of *Scrib* in the dorsal telencephalon induced cortical thickness reduction and alteration of interhemispheric connectivity. In addition, *Scrib* conditional knockout (cKO) mice have behavioral deficits such as locomotor activity impairment and memory alterations. Given *Scrib* broad expression in multiple cell types in the brain, we decided to determine the neuronal contribution of *Scrib* for these phenotypes. In the present study, we further investigate the function of *Scrib* specifically in excitatory neurons on the forebrain formation and the control of locomotor behavior. To do so, we generated a novel neuronal glutamatergic specific *Scrib* cKO mouse line called Nex*-Scrib* ^−/−^ cKO. Remarkably, cortical layering and commissures were impaired in these mice and reproduced to some extent the previously described phenotype in global *Scrib* cKO. In addition and in contrast to our previous results using *Emx1-Scrib* ^−/−^ cKO, the *Nex-Scrib* ^−/−^ cKO mutant mice exhibited significantly reduced locomotion. Altogether, the novel cKO model described in this study further highlights an essential role for *Scrib* in forebrain development and locomotor behavior.

## Introduction

Recent advances in brain development studies have offered novel perspectives into the mechanisms that rule over the pathophysiology of multiple forms of neurodevelopmental disorders (NDDs) and neuropsychiatric disorders that can be associated with rare diseases. For instance, improper neuronal migration can eventually lead to neurobehavioral disorders such as autism spectrum disorders (ASDs) or epilepsy (Pan et al., 2019; Francis and Cappello, 2021). Agenesis of the corpus callosum (ACC) is a condition in which the CC does not develop (De León Reyes et al., 2020) and correlates with many developmental disorders, including ASDs and attention deficit hyperactivity disorders (ADHDs) (Paul et al., 2007; Edwards et al., 2014). Thereby, it appears essential to decipher the mechanisms that govern brain development to further understand the basis of such neurodevelopmental and neuropsychiatric disorders (Cristino et al., 2014; Sullivan and Geschwind, 2019).

Besides its association with cancer, *Scrib* has been implicated in several developmental disorders (Stephens et al., 2018; Bonello and Peifer, 2019; Santoni et al., 2020; Nakajima, 2021). A homozygous *Scrib* spontaneous mouse mutant called *Circletail* (*Crc*) is lethal primarily due to a severe form of neural tube defects (NTDs) (Murdoch et al., 2003)*.* NTDs (OMIM #182940) represent CNS (central nervous system) congenital malformations that affect 1:1,000 children and whose molecular bases are still poorly known (Greene and Copp, 2014; Zohn, 2020)*.* Perturbation of *Scrib* leads to significant planar cell polarity (PCP) defects (Montcouquiol et al., 2003) as tissue polarity coordination is a hallmark of this signaling pathway (Ezan and Montcouquiol, 2013) and its alteration is a driver for NTDs (Murdoch et al., 2014)*.* Studies in various models revealed a broad array of functions for PCP genes in the developing (and adult) nervous system (Tissir and Goffinet, 2013; Sans et al., 2016; Hakanen et al., 2019). The human homolog *SCRIB* is included within a region identified as 8q24.3 that is deleted in patients affected by Verheij syndrome (VRJS/8q24.3 deletion syndrome; OMIM #615583) (Dauber et al., 2013). This locus is associated with ASDs and ADHD (Stam et al., 2009) and is subject to duplication that can lead to profound psychomotor retardation, idiopathic epilepsy, and growth delay (Bonaglia et al., 2005). VRJS is a rare disease with complex characteristics including neurological symptoms such as delayed psychomotor development and epilepsy associated with microcephaly and/or corpus callosum agenesis (Verheij et al., 2009; Dauber et al., 2013). Deletion of *SCRIB* alone has shown partial phenotypes on its own (Wells et al., 2016), and microdeletions were found in children presenting microcephaly, a phenotype recapitulated after independent *Scrib* or *Puf60* knockdown in zebrafish embryos (Dauber et al., 2013). Additionally, our group showed that *Scrib* fine-tunes excitatory synapse, and its mutation leads to disruption of synaptic functions associated with some ASD features (Moreau et al., 2010; Piguel et al., 2014; Hilal et al., 2017). Genome-wide association studies showed *Scrib* duplications, *de novo* mutations, and even mRNA splicing deregulation in some ASD patients (Sans et al., 2016). Although some studies showed alterations in motor neuron migration (Wada et al., 2005), axonal guidance (Walsh et al., 2011), and CNS myelination (Jarjour et al., 2015) after *Scrib* invalidation, its role during early vertebrate brain development—and especially its autonomous and non-autonomous contributions—deserves further investigation.

Our group recently generated brain-specific conditional *Scrib* cKO mouse mutants to assess the impact of the *Scrib* deletion on the brain structure and animal behavior (Ezan et al., 2021). Specifically, we generated *Emx1-Scrib* ^−/−^ cKO and *FoxG1-Scrib* ^−/−^ cKO, leading to a broad and early deletion of *Scrib* throughout the entire dorsal telencephalon (hereafter reported as “global” forebrain *Scrib* cKOs). Their most prominent feature is a marked cortical thickness reduction (microcephaly) and corpus callosum and hippocampal commissure agenesis. This phenotype was correlated with a disruption in various developmental steps of corticogenesis, including neurogenesis, neuronal migration, and axonal connectivity. In addition, we showed that *Emx1-Scrib* ^−/−^ cKO mice have psychomotor deficits such as locomotor activity impairment and memory alterations. Because *Scrib* is broadly expressed in the developing forebrain, including neuronal and glial progenitors, it is essential to characterize which cell types are responsible for *Scrib* implication during brain development and functions.

The goal of the present study is to investigate the specific contribution of neuronal *Scrib* to forebrain formation and to assess its relevance to motor behavior. To do so, we engineered a novel neuronal-specific *Scrib* cKO line called *Nex-Scrib* ^−/−^ cKO mutant mice. We aimed to analyze the consequences of the neuronal-specific depletion of *Scrib* on the structural and behavioral phenotypes and compare this to the previously reported “global” forebrain *Scrib* cKOs, thereby distinguishing abnormalities caused by autonomous *Scrib* (neuronal) versus non-autonomous *Scrib*. Our present results indicate that the late invalidation of *Scrib* selectively in post-mitotic excitatory neurons led to layering defects (indicative of neuronal migration impairment) and corpus callosum hypoplasia, thereby demonstrating a cell-autonomous requirement for *Scrib* during forebrain formation. This selective inactivation of *Scrib* led to an unexpected but marked reduction in locomotion that is opposite to what we previously observed in “global” *Scrib* cKOs. Altogether, our present analysis identified cortical and locomotor defects resulting from the neuronal-specific invalidation of *Scrib*, further revealing an essential role for this gene during forebrain development.

## Results

### Generation of a Novel *Nex-Scrib* ^−/−^ Conditional Knockout Mouse Strain

**FIGURE 1:**
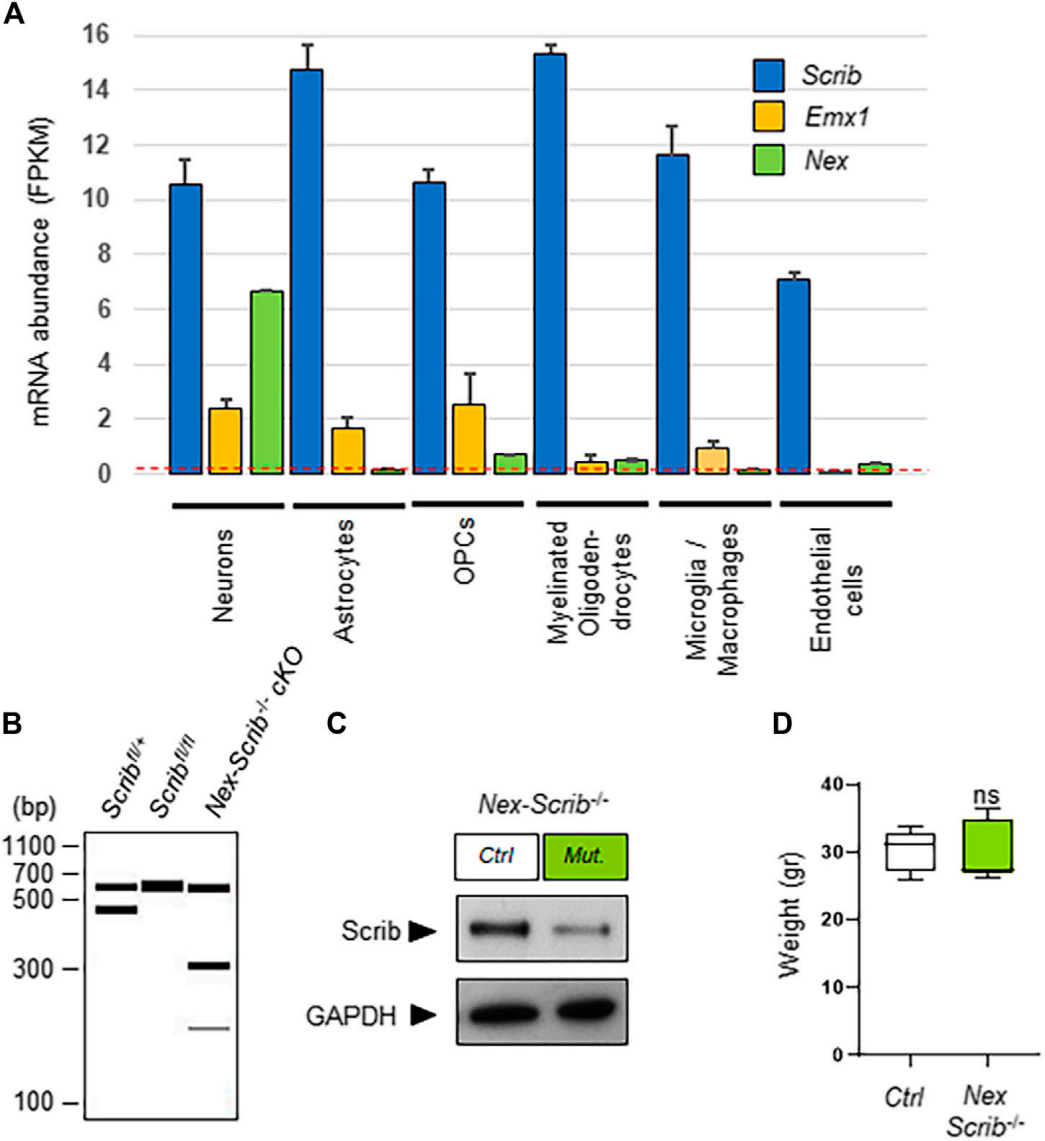
Generation and characterization of a novel neuronal-specific *Scrib* conditional knockout mouse mutant. **(A)** Relative abundances of *Scrib*, *Emx1*, and *Nex* (*NeuroD6*) in different cell types are plotted from the original data set found in the Brain-RNAseq transcriptome database (http://www.brainrnaseq.org) (Zhang et al., 2014). Expression levels are reported as fragments per kilobase of transcript sequence per million mapped reads (FPKM) in various P7 mouse brain cell types [neurons, astrocytes, oligodendrocyte precursor cells (OPCs), myelinating oligodendrocytes, microglia/macrophages, and endothelial cells]. A threshold FPKM value of 0.1 is indicated as a red dotted line. Mean with SEM; *n* = 2. **(B)** Visualization of PCR genotyping results to detect the following alleles: wild-type (437 bp), floxed (541 bp). and targeted cKO (193 bp). The presence of a 300 bp product (lane 3), indicative of a Cre amplification product, confirms the *Cre*-mediated excision in P0 cortices. In the absence of Cre, the wild-type and floxed alleles remain intact as determined by 437 and 541 bp fragments, respectively (lanes 1 and 2). When Cre-mediated recombination occurs (lane 3), the floxed allele is excised, as witnessed by the presence of a 193 bp band. **(C)** Representative western blots showing full-length Scrib protein from the control and *Scrib* ^−/−^ cKO cerebral cortices. Cortical protein extracts of P0 *Nex-Scrib* ^−/−^ cKOs were immunoblotted with the anti-Scrib antibody and anti-GAPDH (control). cKO lysates show reduced levels of Scrib when compared with the control. **(D)** Body weight of 10–11-week-old mice.

We previously reported Scrib expression throughout the embryonic cerebral cortex both in neuronal progenitors and in the radial glia and also in the axonal fibers of the corpus callosum and midline glia at birth (Ezan et al., 2021). To extend these observations, we decided to query *Scrib* gene expression using publicly available cell-specific RNA sequencing data sets (Keil et al., 2018)*.* In order to retrieve more specific information about the cell-type-specific expression patterns of *Scrib*, we reanalyzed the data of an RNA sequencing database (GEO data set GSE52564) of isolated cell types from young adult mouse brains (www.brainrnaseq.org) (Zhang et al., 2014). This data set allowed us to visualize the expression levels of *Scrib* that are expressed in a large variety of cortical cell types, ranging from neurons to astrocytes, oligodendrocyte precursor cells (OPCs), myelinating oligodendrocytes, microglia, and macrophages and also endothelial cells (Figure 1A). As compared with *Nex*, the fairly broad expression of *Emx1* notably in neurons, astrocytes, and oligodendrocytes highlighted the validity of our approach and the necessity to use the *Nex-Cre* strain to specifically delete *Scrib* in neurons (Figure 1A). To specifically evaluate the effects of *Scrib* invalidation in post-mitotic neurons, we generated a novel cKO mouse strain: Scrib^fl/fl^ floxed mice were crossed with Nex-Cre mice to obtain a compound strain called *Nex-Scrib* ^−/−^ cKO mutant mice. Nex-Cre mice express the Cre recombinase at E11.5 in the hippocampus and precursor cells in the sub-ventricular and ventricular zones (SVZ and VZ, respectively), which eventually generate excitatory pyramidal neurons that disperse throughout the cortex (Goebbels et al., 2006; Belvindrah et al., 2007). Most importantly for our study, Cre-mediated recombination in Nex-Cre mice occurs neither in cortical progenitors, glial cells, and oligodendrocytes nor in GABAergic inhibitory interneurons. First, the efficiency of the excision of *Scrib* was validated by polymerase chain reaction (PCR) using DNA samples isolated from the cortex of *Nex-Scrib* cKOs conditional and control littermates (Figure 1B). Second, using a homemade Scrib-specific antibody (Montcouquiol et al., 2006), we performed a western blot analysis to examine to which degree the Scrib product was reduced in the cortex of the *Nex-Scrib* ^−/−^ cKO. As shown by western blot analysis in Figure 1C, *Nex*-Cre-mediated knockout of *Scrib* resulted in a reduction of Scrib protein in the P0 cerebral cortex. General observations of the *Nex-Scrib* ^−/−^ cKO mice indicated that these mice were globally indistinguishable from control littermates and there was no difference in body weight between them (Figure 1D).

### *Scrib* Deletion in Postmitotic Neurons Results in Cortical Layering Defects

**FIGURE 2:**
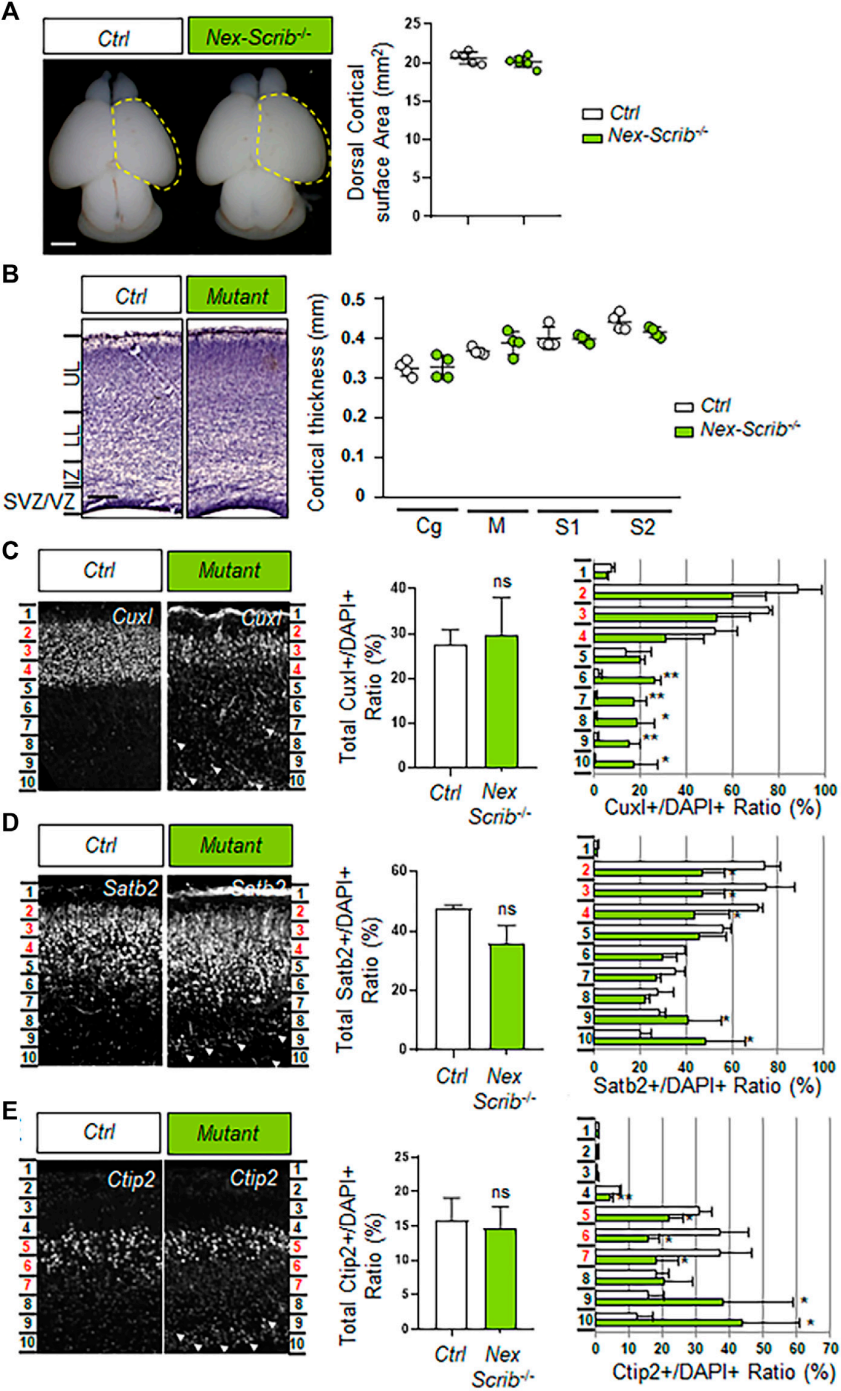
Deletion of *Scrib* specifically in postmitotic pyramidal neurons mostly impacts cortical layering during brain development. **(A)** Dorsal views of P0 *Nex-Scrib* ^−/−^ cKO mouse brains. Cortical plate areas are marked with a yellow dashed line for comparison and quantification. Statistical analysis via a two-tailed *t*-test using at least five brains per genotype from at least three independent experiments. Error bars indicate the SEM. Scale bar: 1 mm. **(B)** Representative hematoxylin staining of coronal sections from the newborn *Nex-Scrib* ^−/−^ cKO motor cortex at the caudal level and its control. No significant reduction of the caudal motor cortex thickness was observed in the mutant. Cortical plate thickness was measured radially from the top of the upper layer (UL) to the bottom of the lower layer (LL) of the cortex. IZ: intermediate zone, SVZ: subventricular zone, VZ: ventricular zone. Statistical analysis via a two-tailed *t*-test using at least four measurements per genotype from at least three independent experiments. Error bars indicate the SEM. Scale bar: 0.2 mm. **(C–E)** Representative immunofluorescence staining of CuxI **(C)**, Satb2 **(D)**, and Ctip2 **(E)** on coronal sections from newborn *Nex-Scrib* ^−/−^ cKO brains (green) and the control in the caudal motor cortex. Quantification of CuxI-, Satb2-, and Ctip2-positive neurons is shown as a percentage of total DAPI-positive cells (not shown) for each ROI (see methods). In each ROI, layers II–III (Cux1- and Satb2-positive) correspond to bins 2–4, while layer V (Ctip2-positive) corresponds to bins 5–7 (highlighted in red in every figure). Migration defects are suggested by the fact that several ectopic CuxI-, Satb2-, and Ctip2-positive cells are mislocalized in lower bins, namely, bins 8–10 (white arrowheads). Statistical analysis via a two-tailed *t*-test (*p < 0.05, **p < 0.01) using between three and four measurements per genotype from at least three independent experiments. Error bars indicate the SD.

We next investigated whether the cerebral cortex of *Nex-Scrib* ^−/−^ cKO mutants was impaired. We observed that the overall brain structure and particularly the dorsal surface areas of the cerebral hemispheres of the *Nex-Scrib* ^−/−^ cKO mutants appeared normal when compared to their control littermates (Figure 2A). Furthermore, an histological analysis showed no obvious reduction of cortical thickness throughout the cingulate (Cg), motor (M), primary somatosensory (S1), and secondary somatosensory (S2) in *Nex-Scrib* ^−/−^ cKO mutants versus the control (Figure 2B). We next performed an immunohistological analysis in *Nex-Scrib* ^−/−^ mutant mice motor cortices to assess if the cortical layering is impaired in these mutants. We selected antibodies directed against specific markers of callosal projection neurons (CPNs) such as CuxI and Satb2 (late-born neurons from layer II/III) and Ctip2 (earlier-born neurons from layer V) as some of these markers are essential to the formation of the corpus callosum (Alcamo et al., 2008; Britanova et al., 2008). This analysis revealed no major CPN production defects (see central histograms in insets in Figures 2C–E). However, the distribution of both early- and late-born neurons was affected with many CPNs mislocalized in deeper layers of the cortex at P0 (see arrowheads in Figures 2C–E). These defects in layering are exemplified by the quantification of markers in bin 10 (deepest bin), showing an increase in CuxI (ctrl, 0.6% ± 0.1; mutant, 17.3% ± 10.8; *p* = 0.046) (Figure 2C), Satb2 (ctrl, 20.3% ± 4.6; mutant, 48.6% ± 17.3; *p* = 0.04) (Figure 2D), and Ctip2 percentages (ctrl, 12.2% ± 5.1; mutant, 43.7% ± 17.3; *p* = 0.04) (Figure 2E). The fact that both early-born Ctip2-positive CPNs and also late-born CuxI- and Satb2-positive CPNs cannot reach their proper layer indicates that neuronal *Scrib* is essential at a cell-autonomous level during radial cortical migration.

### Corpus Callosum Hypoplasia in *Nex-Scrib* ^−/−^ cKO Mutants

**FIGURE 3:**
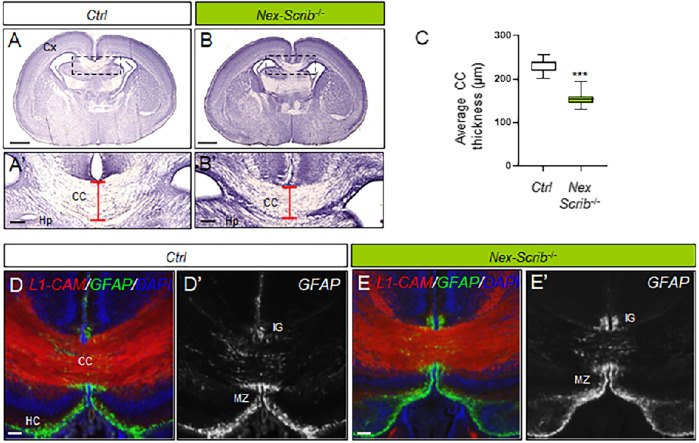
Corpus callosum dysgenesis in Nex-Scrib^-/-^ cKO mutants. **(A,B)**, Representative hematoxylin staining of coronal sections from newborn Nex-Scrib^-/-^ cKO brains **(B)** and control brains **(A)** at the caudal level. Dashed boxes in **(A,B)** are magnified in **(A’,B’)**. Scale bar: 1 mm. **(A’,B’)**, Higher magnification for selected insets (boxed areas) from **(A,B)** illustrating the reduction of corpus callosum thickness in the Nex-Scrib^-/-^ cKO brains (see red brackets). Cx: Cortex, Hp:Hippocampus. Scale bar: 0.1 mm. **(C)**, Average thickness of the corpus callosum in μm in newborn Nex-Scrib^-/-^ cKO brains (green) and control brains (white). Statistical analysis via a two-tailed t-test (***p<0.01) using respectively 10 and 9 measurements per genotype from 3 independent experiments. Error bars indicate the SD. **(D-E’)**, Representative immunofluorescence staining of L1-CAM (red) and GFAP (green) as a merged image **(D,E)** or GFAP only [**(D’,E’)**, gray] on coronal sections from newborn Nex-Scrib^-/-^ cKO brains [**(E,E’)**, green] and their respective controls **(D, D’)** at the caudal level. GFAP-positive midline glia structures do form normally in Nex-Scrib^-/-^ cKO mutants despite CC hypoplasia. Scale bar: 0.1 mm.

Our previous results in global brain *Scrib* cKO showed a severe caudal agenesis of the corpus callosum and dorsal hippocampal commissure associated with Probst bundles which we attributed mostly to midline glial defects (Ezan et al., 2021). To assess if this phenotype could also have a neuronal component, we performed histological analysis on newborn *Nex-Scrib* ^−/−^ mutants (Figures 3A–B’). Coronal histological sections show a marked reduction in the size of the corpus callosum (CC hypoplasia), that is, ∼35% thinner in newborn *Nex-Scrib* ^−/−^ cKO mutants relative to their control littermate (Figure 3C). As expected, immunostaining of P0 coronal sections using antibodies against GFAP (marker of midline glia) and the axonal marker L1-CAM revealed that late deletion of *Scrib* resulted in a normal organization of the midline glia structures, with GFAP-positive cells positioned in proper locations, surrounding the CC, as in control littermate coronal sections (Figures 3D–E’). These findings indicate that *Scrib* deletion of in the dorsal telencephalon of *Nex-Scrib* ^−/−^ cKOs (including the cerebral cortex and the hippocampus) has a neuronal-specific contribution on the formation of commissures such as the corpus callosum. Taken as a whole, we show that neuronal *Scrib* is essential for CPN migration and proper cerebral cortex layering and thus in corpus callosum formation.

### Locomotor Activity Is Decreased in *Nex-Scrib* ^−/−^ cKO Mutant Mice

**FIGURE 4:**
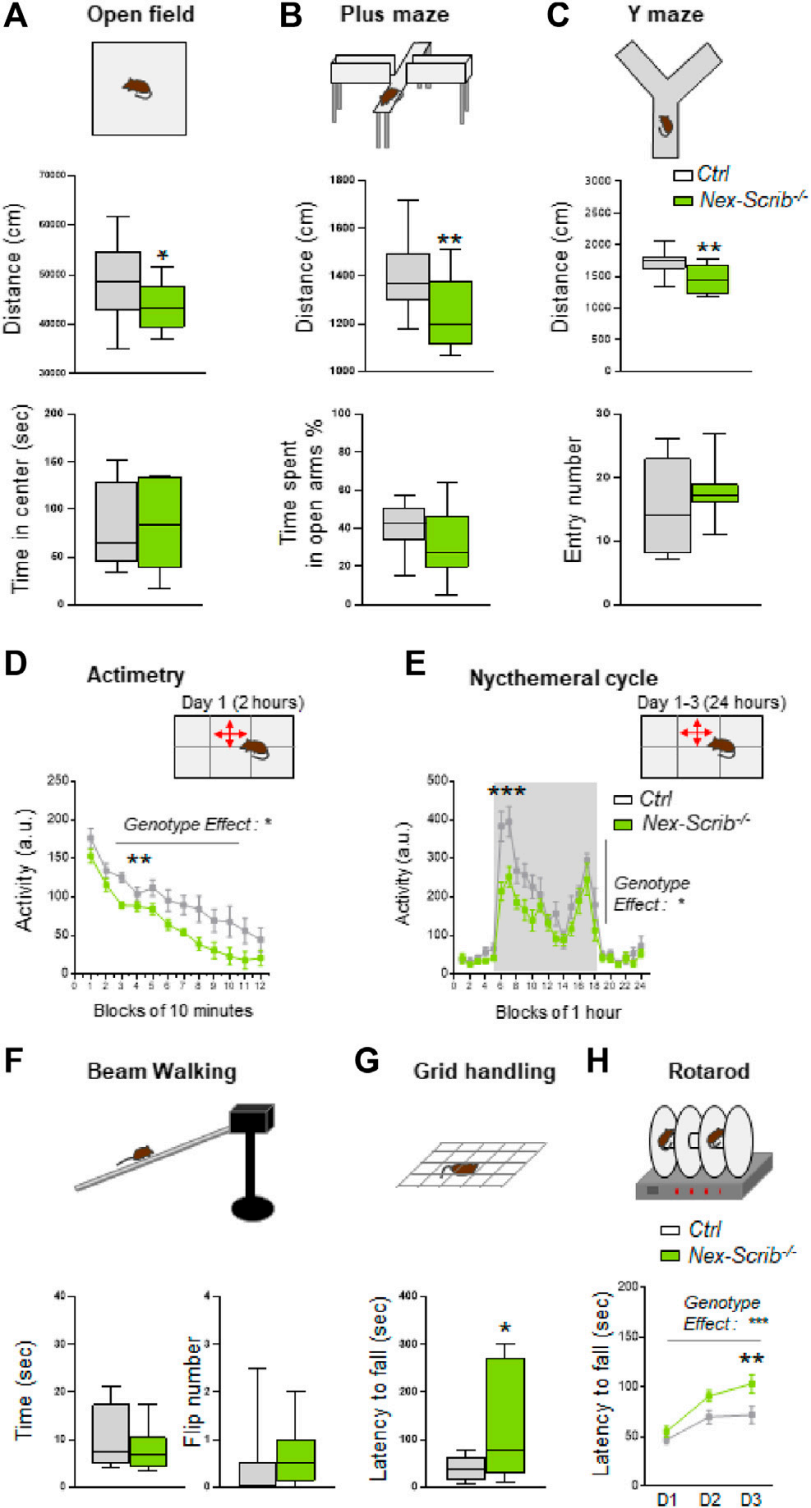
*Nex-Scrib* ^−/−^ cKO mice display decreased locomotor activity and no impairment of motor coordination. **(A–H)** Behavioral responses in adult *Nex-Scrib* ^−/−^ cKO mice (green) as compared with their littermate control (white). Spontaneous locomotor activity was monitored in several assays: in the open field test **(A)**, in the elevated plus maze test **(B)**, in the Y-maze **(C)**, during exposure to a novel home cage **(D)**, and during the nycthemeral cycle **(E)**. *Nex-Scrib* ^−/−^ cKO mice travel shorter distances than their respective controls. The circadian profile of the locomotor activity was measured in 1 h increments during 24 h under 12 h light (blocks 18–6) and 12 h dark conditions (blocks 6–18). **(F–H)** Motor coordination and balance. In the beam walking, grid handling, and rotarod tests, no defects were observed in *Nex-Scrib* ^−/−^ mice. Values are presented at means ± s.e.m. from 6–14 mice per genotype. (*p < 0.05, **p < 0.01) versus the control littermate group.

To assess behavioral alterations in adult *Nex-Scrib* ^−/−^ mice and their littermate controls (10–20 weeks), we performed tests of anxiety-like behavior and locomotor and exploratory activity. We showed that *Nex-Scrib* ^−/−^ mice exhibited significantly reduced exploration characterized by a reduction of the followed distance in the open field (*t*-test; t25 = 2.13, *p* < 0.05; Figure 4A) in the elevated plus-maze (*t*-test; t24 = 2.55, *p* < 0.01; Figure 4B) and in the Y-maze (*t*-test; t24 = 3.09, *p* < 0.01; Figure 4C). The *Nex-Scrib* ^−/−^ mice do not display changes in anxiety-like behavior as reflected by the time spent at the center of the open field (*t*-test; t25 = 0.41, *ns*; Figure 4A) and the time spent in the open arms of the elevated plus-maze (*t*-test; t24 = 1.38, *ns*; Figure 4B). In addition, the measure of spontaneous activity in a novel home cage showed *Nex-Scrib* ^−/−^ mice that were significantly less active than their controls (genotype effect: F1,143 = 6.36, *p* < 0.05; Figure 4D). The same hypoactivity was observed during the nycthemeral cycle in their home cages as determined by 24-h continuous monitoring of locomotor activity (genotype effect: F1,13 = 7,579, *p* < 0.05; Figure 4E). Since motor coordination and balance are essential to normal motor functions, we have submitted mice to the beam walking, grid handling, and rotarod tests. No defects were observed in *Nex-Scrib* ^−/−^ mice in terms of balance measured by the time spent on the beam (*t*-test; t14 = 0.33, *ns*; Figure 4F) and the number of paw slips during walking (Mann–Whitney test, *ns*; Figure 4F). Also, the motor coordination is not impaired as mice take longer to fall in the grid handling test (*t*-test; t15 = 2.19, *p* < 0.05; Figure 4G) and the rotarod (genotype effect: F1,81 = 12.88, *p* < 0.001; Figure 4H). These findings demonstrate that *Nex-Scrib* ^−/−^ mutant mice are hypoactive in novel and familiar environments, in contrast to *Emx1-Scrib* ^−/−^ mutant mice (Ezan et al., 2021). Taken together, our results point toward an important role for *Scrib* in the telencephalic glutamatergic circuitry in mouse locomotor behavior.

## Discussion

In the present study, we investigated the neuronal-specific contribution of *Scrib* on mouse embryonic brain development and locomotor behavior. We successfully developed and characterized a novel mouse cKO strain where *Scrib* is deleted only in postmitotic excitatory pyramidal neurons throughout the telencephalon referred to as the *Nex-Scrib* ^−/−^ cKO line. We show that neuronal *Scrib* is essential during corticogenesis and corpus callosum formation. Unexpectedly, our behavioral analysis showed a decreased locomotor activity in *Nex-Scrib* ^−/−^ cKO, which is a noticeable distinction from our observations with *Emx1-Scrib* ^−/−^ mutant mice, and our results are compiled for comparison in Table 1. Overall, the lack of *Scrib* expression in cortical glutamatergic neurons triggers a cascade of neuroanatomical cortical defects that ultimately negatively impact the formation of a neuronal circuit that is essential to mouse locomotor behavior.

**TABLE 1:** Comparison of anatomical and behavioral phenotypes in different *Scrib* conditional Ko mice strains.

**Genotype**	**Nex-Scrib-/-cKO**	**Emx-Scrib-/-cKO**	**FoxG1-Scrib-/-cKO**
Reference	Present study	Ezan et al. (2021)	Ezan et al. (2021)
Spatio-temporal invalidation characteristics	Dorsal Telencephalon	Dorsal Telencephalon	Dorsal and ventral Telencephalon
	Starting E11.5	Starting E10.5	Starting E8.5
	Post-mitotic excitatory glutamatergic neurons	- Cortical and hippocampal progenitors	- Cortical and Hippocampal progenitors and ganglionic eminence progenitors
		
**Phenotype**			
**Brain anatomy**	**Impaired**	**Impaired**	**Major impairment**
Dorsal cortical area	normal	decreased	strongly decreased
Cortical thickness	normal	decreased	strongly decreased
Cortical layering	impaired	impaired	strongly impaired
Corpus Callosum	impaired (hypoplasia)	Impaired(rostral)	Impaired(rostral/caudal)
Hippocampal commissure	impaired (hypoplasia)	Impaired(rostral)	Impaired(rostral/caudal)
midline glia positioning	normal	Impaired(rostral)	Impaired(rostral/caudal)
**Locomotor activity**	**Decreased**	**Increased**	**Not tested**
Open Field	decreased	increased	Not tested
Plus maze	decreased	increased	Not tested
Y-maze	decreased	increased	Not tested
Actimetry	decreased	increased	Not tested
Nycthemeral activity	decreased	increased	Not tested
**Anxiety**	**Normal**	**Normal**	**Not tested**
Open Field	normal	normal	Not tested
Plus maze	normal	normal	Not tested
**Motor coordination**	**Increased**	**Normal**	**Not tested**
Beam walking	Normal	normal	Not tested
Grid handling	Increased	normal	Not tested
Rotarod	Increased	normal	Not tested

### Cell-Autonomous Contribution of *Scrib* in Cortical Pyramidal Postmitotic Neurons Plays a Prominent Role in Their Cortical Positioning

Our present characterization of the *Nex-Scrib* ^−/−^ cKO line revealed that *Scrib* is essential to cortical layering. While *Scrib* is inactivated in cortical progenitors, in radial glial cells, in *Emx1-Scrib* ^*−/−*^ mutant mice, the ablation of *Scrib* solely in postmitotic cortical neurons, during cortical development stages, led to significant neuronal migration defects. Indeed, *Scrib* loss in neurons did affect their ability to migrate into adequate cortical layers, consistent with our observations in *Emx1-Scrib* ^*−/−*^ cKO (Ezan et al., 2021). More specifically, we observed cortical layering defects in *Nex-Scrib* ^−/−^ cKO brains with mispositioned neurons from layers II/III and V. Both early-born Ctip2-positive CPNs and also late-born CuxI- and Satb2-positive CPNs are impacted, merely by their numbers, but rather by their positioning, implying a prominent role for *Scrib* throughout every cortical layer. Notably, the extent of these layering defects is similar to the one observed in *Emx1-Scrib* ^*−/−*^ cKO mutants (Ezan et al., 2021), indicating that *Scrib* contribution to the neuronal radial migration relies primarily, if not totally, on its cell-autonomous neuronal contribution. Notably, none of the *Nex-Scrib* ^−/−^ cKO mice suffered from microcephaly, and this was expected considering that the main characteristic of this cKO line is to bypass the negative impact of *Scrib* loss on proliferation and neurogenesis observed in the *Emx1-Scrib* ^*−/−*^ mutant mice (Ezan et al., 2021). At present, the precise molecular mechanisms at play in *Scrib*-dependent neuronal migration remain elusive (Jossin, 2020) and are challenging to define owing to the complex interplay of neurons with radial glia in the cerebral cortex (Marin et al., 2010; Hansen and Hippenmeyer, 2020). However, coordinated regulation of adhesion forces between neurons and radial glia is required for proper neuronal migration, and we suspect that *Scrib* may be central to this process as shown in another context (Qin et al., 2005).

### Postmitotic *Scrib* has an Impact on Interhemispheric Commissure Formation

In the present study, we show that the neuronal-specific invalidation of *Scrib* leads to a partial but marked corpus callosum hypoplasia (reduction of CC in thickness)/dysgenesis. We can infer that these commissural defects stem from the CPN migration defects observed in the *Nex-Scrib* ^−/−^ cKO mice: while some CPNs do reach their proper cortical layer, the others are unable to correctly send their axonal projections toward the contralateral side as observed in many other models with partial ACC (Edwards et al., 2014). As expected, *Scrib* loss solely in neurons does spare midline glial structures that are essential to promote interhemispheric tissue fusion and remodeling (Yu et al., 2010; Gobius et al., 2016), and the few callosal fibers reaching the midline can ultimately cross it. Tissue fusion is a central process during neural tube closure (Pai et al., 2012) and during interhemispheric remodeling in CC formation (Wahlsten et al., 2006; Paul et al., 2007), and we suspect that *Scrib* is involved in this process in other tissues as well (see below). Our previous study indicated that the broad loss of *Scrib* expression in the midline glia leads to the disorganization of that structure (Ezan et al., 2021). The formation of Probst bundles (Shu et al., 2003) in *Emx1-Scrib* ^−/−^ mutant mice suggested that a non-autonomous *Scrib*-dependent mechanism affects the guidance and the crossing of the commissural axons (Ezan et al., 2021). Supporting this hypothesis, the partial corpus callosum dysgenesis and the proper midline glia architecture we observe in the *Nex-Scrib* ^−/−^ cKO mice are in favor of an additional requisite contribution of *Scrib* in glial cells for a complete CC formation and hence the strong ACC phenotype we observe in our global *Scrib* cKO models. Interestingly, *Scrib* is enriched in oligodendrocytes (where *Emx1* is expressed) and is involved in the myelination initiation (Jarjour et al., 2015). However, oligodendrocyte-specific deletion of *Scrib* does not appear to impact CC formation. Also, further experiments using conditional approaches to inactivate *Scrib* in glial subpopulations by different means would help clarify this issue.

Overall, our observations using complementary *Scrib* cKO models indicate that it has a major role in cortex development and commissure formations on a large spatio-temporal scale. While *Scrib* loss in *Emx1-Scrib* ^*−/−*^ mutant mice displays major microcephaly and complete CC agenesis, *Nex-Scrib* ^*−/−*^ mutant mice display “only” cortical layering defects and partial CC dysgenesis. An explanation is that the time course and the extent of the genetic modification in the latest model were insufficient to fully impact these processes. In our previous investigation, *Scrib* ^*fl/fl*^ floxed mice were crossed with *FoxG1*-Cre or *Emx1*-Cre mice to obtain *FoxG1-Scrib* ^−/−^ and *Emx1-Scrib* ^−/−^ cKO mutant mice, respectively, with an early *Scrib* excision in different cell types in the brain (see Table 1). Cre-mediated recombination in *FoxG1*-Cre mice starts as early as E8.5 and occurs mainly in all cells from both dorsal and ventral telencephalons (Hébert and McConnell, 2000). This includes neuroepithelial progenitors that specify various neuronal and glial cells from the neocortex and hippocampus and also ganglionic eminence progenitors giving rise to GABAergic interneurons. In *Emx1*-Cre mice, the Cre recombinase is expressed as early as E10.5 and only in the dorsal telencephalon (Gorski et al., 2002). *Emx1*-Cre expression led to *Scrib* excision of most neurons of the neocortex and hippocampus in glial cells of the pallium but not in the GABAergic interneurons. While these global *Scrib* cKOs clearly define a role for *Scrib* in normal forebrain formation, they do not distinguish between a neuronal and non-neuronal site of action. Also, it could be argued that the partial phenotype observed in *Nex-Scrib* ^−/−^ cKO mice is presently due to a subspecific invalidation of *Scrib*. The absence of Probst bundles in *Nex-Scrib* ^−/−^ cKO mice suggests that neuronal *Scrib* is merely implicated in axonal guidance during CC formation and *Scrib* impact on this process may rely on other cell types (e.g., glial cells). A requirement for *Scrib* in axonal guidance has indeed been shown at the optic chiasm (Rachel et al., 2000) and during spinal commissure formation in zebrafish (Sun et al., 2016). Alternatively, given its essential role in many cellular processes, another consideration is that the lack of neuronal *Scrib* is functionally compensated by other LAP (leucine-rich repeats and PDZ) family members (Santoni et al., 2002), as recently evidenced during apicobasal polarity establishment in mammalian epithelial cells (Choi et al., 2019).

### *Scrib* Deficiency During Embryogenesis in the Excitatory Circuitry Leads to Locomotor Disabilities in Adult Animals

From the behavioral standpoint, we observed in the present study a hypoactive locomotor behavior phenotype in *Nex-Scrib* ^−/−^ cKO mice in every paradigm tested (Plus maze, Open field, Y-maze). This observation is quite remarkable considering that our previous analysis using the *Scrib* cKO mice revealed an opposite phenotype in locomotor behavior (Ezan et al., 2021) (see Table 1). Specifically, we observed in *Emx1-Scrib* ^−/−^ mutant mice a hyperactive locomotor behavior comparable to established models (Itohara et al., 2015). Surprisingly, *Scrib* signaling solely in glutamatergic neurons not only proved to convey an opposite effect on locomotion compared to *Emx1-Scrib* ^*−/−*^ mutant mice but also seems to be involved in the regulation of motor coordination. These results strongly support a critical role for *Scrib* in regulating locomotor behavior of the animal and also highlight a spatial and temporal dependence on *Scrib* regarding this regulation. To ensure direct comparison to our previous study, our present work is limited to male mice. A comparative study in female mice is warranted to evaluate if the deletion of *Scrib* in glutamatergic neurons would also result in reduced locomotion. Because of the early and broad expression of *Emx1* in the brain, it is impossible to identify what specific circuit drives the hyperactive locomotor behavior. On the other hand, *Nex* expression is restricted to postmitotic glutamatergic neurons, supporting the hypothesis that the pathological origin for the hypoactive locomotor behavior has a strict neuronal origin. In addition, since *Nex-Cre;Rosa26* reporter strains show some expression in the cerebellum (Goebbels et al., 2006; Belvindrah et al., 2007), where *Scrib* is expressed (Moreau et al., 2010), we cannot exclude the possibility that some effects that we observe in our tests are due to an impact of a loss of *Scrib* expression in the cerebellum. Importantly, locomotor behavior is unaffected in *CamK-Scrib* ^*−/−*^ mutant mice that target postnatal excitatory neurons of the cortex and the hippocampus (Hilal et al., 2017), demonstrating that neuronal *Scrib* signaling is pivotal for the locomotor output essentially during embryonic brain development. Thus, these findings support the relevance of this gene in glutamatergic circuits to control the excitatory drive on locomotor behavior, a previously unknown function of *Scrib* on behavioral performance. It is presently unclear how *Scrib* could select the cell-type type in which they exert these opposing effects, but we can hypothesize that this happens through the regulation of glutamatergic signaling (Piguel et al., 2014). Given the pronounced locomotor dysfunction in *Scrib* cKO mice, which is also altered in *Nex-GluN2B* cKO (Wang et al., 2011), deficiencies in *Scrib* expression could contribute to the impairment of the cortical excitatory/inhibitory balance (Nelson and Valakh, 2015). In the control situation, Scrib helps to maintain a stable glutamatergic activity at the neuronal circuit level, possibly through the regulation of GluN2B subunits as has been shown by us previously in other contexts (Piguel et al., 2014), thereby representing an important regulator of the motor cortex. An activity-related control mechanism of the glutamatergic signaling is removed when deleting *Scrib* from cortical/hippocampal glutamatergic neurons. Consequently, the excitability of the whole circuit is unbalanced in an opposite manner in *Nex-Scrib* ^−/−^ cKO and *Emx1-Scrib* ^*−/−*^ cKO mutant mice, but which both represent a deleterious state, leading to locomotor behavioral alterations. In the future, it would be of interest to survey VRJS patients in clinical studies that may report dysfunctions in locomotion and general activity. Altogether, these findings underline the complexity and multi-faceted nature of *Scrib* in the forebrain and emphasize the requirement for additional cell-specific investigations in parallel with global brain experimentations.

### Multiple Features of VRJS Are Recapitulated After *Scrib* Loss

Original reports for VRJS described patients with dysmorphic facial features, cardiac ventricular septal defects, coloboma, and skeletal abnormalities together with neurological features including delayed psychomotor development, recurrent seizures, CC agenesis, and microcephaly (Verheij et al., 2009; Dauber et al., 2013). The minimal common deletion encompassed the *SCRIB* and *PUF60* genes whose invalidation in zebrafish showed their contribution to VRJS clinical features (Dauber et al., 2013). Our previous study (Ezan et al., 2021) and the present one support a role for *Scrib* in some brain structural deficits observed in VRJS. A case with 8q24.3 deletion including *SCRIB* showed dysmorphic facial features, cardiac defects, and vertebrate anomalies but no microcephaly, highlighting the complexity of human genetics (Wells et al., 2016). Other data suggest that *Scrib* may also be implicated in extra-neurological features of VRJS. *Scrib* mutant *Crc* and myocardium-specific *Scrib* cKOs display ventricular septal defects and thinned myocardial walls that recapitulate cardiac features of VRJS patients (Phillips et al., 2007; Boczonadi et al., 2014) in line with a fetal case carrying *SCRIB* haploinsufficiency (Wells et al., 2016). Also, an exome analysis identified a rare *SCRIB* variant that resulted in coloboma (Hocking et al., 2018). A common denominator to the *Scrib*-associated defects such as NTDs, ACC, ventricular defects, or coloboma is the failure in the tissue fusion process (Yu et al., 2010; Hocking et al., 2018). Finally, in the light of the growing interest for an ontogenetic link between the nervous system and craniofacial development, it is worth mentioning the implication of PCP signaling to the latest (Topczewski et al., 2011). A role for *SCRIB* in the face, vertebrae, and digit development was suggested (Wells et al., 2016), and *Scrib* expression is increased in a craniosynostosis chick embryo model (Li et al., 2011) and also in mature osteoclast *in vitro*, hinting at a function in bone mass regulation (Orriss et al., 2018). At present, an implication of *SCRIB* in craniofacial defects may appear provocative, but these data further warrant investigation on its role in craniofacial and skeletal development and its relationship toward brain formation (Motch Perrine et al., 2017).

### Concluding Remarks

Taken as a whole, our results summarized in Table 1 show that 1) various neuroanatomical effects of *Scrib* are conducted by different cellular circuits, which genetic approaches can dissect; 2) cortical glutamatergic neurons mediate a large portion of the cell-autonomous deleterious effects of *Scrib* on neuronal migration and thus cortical layering; 3) glutamatergic neurons do partially mediate the effects of *Scrib* on global cerebral cortex formation and corpus callosum formation; 4) *Scrib* expression in cortical glutamatergic neurons plays a pivotal role in mediating the hypolocomotor effects observed in *Nex-Scrib* ^−/−^ cKO mice. Together with our previous study, our present work suggests a dysfunction in the telencephalic neuronal circuitry in behaving *Scrib* ^−/−^ cKO mice that ultimately points to a dysregulation of the excitation/inhibition balance as a potential mechanism for locomotor dysregulation in our models. This neuroanatomical and behavioral integrative study further strengthens our perception of the importance of Scrib in brain formation relevant to neurodevelopmental diseases such as the rare Verheij/8q24.3 deletion syndrome.

## Materials and Methods

### Animal Care and Use

Mice were housed in the animal facility of the Neurocentre Magendie under controlled conditions (lights on from 7:00 a.m. to 7:00 p.m.) with *ad libitum* food and water. For timed pregnancy, detection of the vaginal plug assessed in the morning was designated as embryonic day E0.5. This study was carried out in compliance with the ARRIVE guidelines and according to the European Directive 2010/63/EU. It was approved by the Bordeaux University Animal Care and Use Committee (#5012016-A and #5012015-A).

### Generation of the Neuronal-Specific *Scrib* Conditional Knockout

*Nex*-Cre (also called *Neurod6*-cre) mice were obtained from Klaus-Armin Nave (Max Plank Institute, Germany). *Scrib* ^*fl/fl*^ floxed mice were generated previously in collaboration with Neal Copeland, Nancy Jenkins, and Rivka Rachel at NIC/NIH (Yamben et al., 2013). The *Scrib* mouse gene contains 38 exons that are translated into a full-length 180 kDa protein. In *Scrib* ^*fl/fl*^ floxed mice, lox-P sequences are inserted before exon 2 and after exon 8 (Hilal et al., 2017). All the exons between 2 and 8 are excised after recombination, leading to the loss of the full-length protein (Ezan et al., 2021). Heterozygous rodents were intercrossed to generate homozygous and wild-type littermates as previously published (Ezan et al., 2013)*.* Specifically, heterozygous male Nex^Cre/+^; *Scrib* ^*fl/fl*^ mice were intercrossed with female *Scrib* ^*fl/fl*^ mice to produce offspring of two genotypes, with or without the Nex^Cre^ transgene: Nex^+/+^; *Scrib* ^*fl/fl*^ (which is the control littermate, Ctrl) or Nex^Cre/+^; *Scrib* ^*fl/fl*^ (referred to as *Nex-Scrib* ^−/−^). *Nex-Scrib* ^−/−^ mutant mice are viable and fertile and do not display any gross physical abnormalities. *Nex*-Cre mice express the Cre recombinase at or before E12.5 in the hippocampus and in precursor cells in VZ/SVZ, which eventually generate pyramidal neurons that disperse throughout the cortex (Goebbels et al., 2006; Belvindrah et al., 2007). It is also expressed in a scattered pattern in the cerebellum (Goebbels et al., 2006; Belvindrah et al., 2007). Of note, Cre-mediated recombination in *Nex*-Cre mice occurs neither in glial cells and oligodendrocytes nor interneurons. Genotyping was performed on cortex samples by PCR using the following primers: F (5′-gca​cac​tgg​gta​tca​tgg​cta-3′), R1 (5′-gca​atc​tcc​aga​gcc​tta​cag​a-3′), and R2 (5′-ccc​ttg​gaa​acc​tac​atc​cca​a-3′) (Ezan et al., 2021). Wild-type (WT), floxed, deleted *Scrib* alleles were distinguished by the following amplified products: for the WT band (F + R1; 437 bp), flox band (F + R1; 541 bp), and cKO band (F + R2; 193 bp) (Figure 1B). Cre genotyping was performed using the following primers: F (5′-cgg​cat​ggt​gca​agt​tga​ata-3′) and R (5′-gcg​atc​gct​att​ttc​cat​gag-3′), resulting in a 300 bp band. PCR product analysis was done using a Labchip GX microfluidic electrophoresis system (Perkin-Elmer) using the DNA5k kit and visualized by a virtual gel image generated by the Labchip GX software. Cre minus littermates showed no detectable phenotype and were thus used as controls.

### Western Blot

Cerebral cortices of P0 pups were homogenized in RIPA buffer [10 mM Tris-Cl (pH 8.0), 1 mM EDTA, 0.5 mM EGTA, 1% Triton X-100, 0.1% sodium deoxycholate, 0.1% SDS, 140 mM NaCl] supplemented with protease inhibitor cocktail (Complete™; Roche). Using the Pierce BCA Protein assay kit (Thermo Scientific), we determined the protein sample concentration and equal amounts of protein were diluted in the sample buffer, separated by sodium dodecyl sulfate–polyacrylamide gel electrophoresis (SDS-PAGE) and visualized using the enhanced chemiluminescence (ECL) as previously described (Ezan et al., 2021). Protein extracts were separated on an 8% gel and transferred overnight to PVDF membranes (Millipore). After blocking with 5% non-fat milk in 1×Tris-buffered saline pH 7.4; 0.05% Tween-20 (TBS-T) for 30 min at room temperature (RT), membranes were first incubated with homemade rabbit anti-Scrib ab (MM468; 1:5,000) (Montcouquiol et al., 2006) and mouse anti-GAPDH (Millipore; 1:5,000) for 1 h at RT and then with secondary antibodies (Amersham; donkey anti-rabbit or anti-mouse IgG conjugated to horseradish peroxidase, 1:20,000) in 1% non-fat milk in TBS-T for 1 h at RT. Immunoreactive signals were detected using the Pierce ECL substrate (Thermo Scientific). Band intensity analysis was performed by densitometry with the ImageJ software (http://imagej.nih.gov), and band intensity was then quantified as a percentage of control band intensity using a representative of at least three independent experiments.

### Histology and Immunofluorescence

For histology, brains (P0 new-born) were harvested and fixed in Bouin’s fixative (Electron Microscopy Sciences) overnight as previously described (Ezan et al., 2021). Brains were dehydrated in ethanol, paraffin-embedded, and sectioned coronally (20 µm); then, sections were collected onto Superfrost plus Gold slides (Thermo Scientific) and stained with hematoxylin and mounted with Entellan (Millipore). The brains sections were examined under a Leica MZ-16 stereomicroscope, imaged using the NanoZoomer 2.0-HT slide scanner in the Bordeaux Imaging Center (http://www.bic.u-bordeaux.fr) and finally analyzed using the NDP viewer software (Hamamatsu).

For immunofluorescence staining (IF), experiments were performed as previously described in Ezan et al., 2021. P0 pups were anesthetized with pentobarbital and perfused transcardially with 4% paraformaldehyde (PFA) in PB buffer (0.1% PBS, 0.9% NaCl, PH = 7.4). After postfixation in 4% PFA overnight at 4°C, dissected brains were infused in 30% sucrose in PB overnight and embedded in O.C.T (Sakura Finetek). Brains were cryosectioned coronally (20 μm thickness) and mounted on Superfrost plus slides (VWR). Sections were PBS-hydrated, permeabilized with 0.2% Triton-X100/PBS (PBS-T), blocked using 10% Normal Goat Serum (NGS), and incubated overnight with the following primary antibodies in 5% NGS overnight at 4°C: rat Ctip2 (Abcam; #ab18465, 1:500), mouse Satb2 (Abcam; #ab51502, 1:100), rabbit Cux1 (Santa Cruz Biotechnology; #sc-13024; 1:100), rabbit anti-GFAP (DAKO; #Z0334; 1:1,000), and rat anti-L1-CAM (Millipore; #MAB5272; 1:1,000). After three washes in PBS-T, samples were incubated for 2 h with the secondary antibody Alexa Fluor^®^ 488 or 546 Goat Anti-Mouse/Rat/Rabbit IgG (Life Technologies; 1:200) and then with 4,6-diamidino-2-phenylindole (DAPI) (Life Technologies; 1:20,000) for 30 min. Finally, after three PBS washes, samples were mounted with the Prolong Gold anti-fading reagent (Invitrogen). Immunostained sections were imaged using a Zeiss Axio Imager Z1 microscope and Axio Vision (Version 4.7) imaging analysis software (Carl Zeiss). Images were processed with Photoshop CS5 software (Adobe) and ImageJ software (http://imagej.nih.gov).

### Quantitative Analysis of the Laminar Position in the Cerebral Cortex

Sections chosen for analysis were matched along the rostral-caudal axis and observed at the caudal level for every sample. After conversion to gray values and normalization to background staining, each region of interest (ROI) in the motor cortex was subdivided into 10 equal bins from the pia (bin 1) to the outer border of the intermediate zone (bin 10) to assess CPN distribution. Doing so, layers II–III (Cux1- and Satb2-positive) were corresponding to bins 2–4, while layer V (Ctip2-positive) was corresponding to bins 5–7. The number and distribution of CuxI-, Satb2-, Ctip2-, and DAPI-labeled cells in each zone were determined manually using the cell counter plugin for ImageJ (http://rsbweb.nih.gov/ij/plugins/cell-counter.html) and blindly to the genotype. Data are given as a ratio (in percent) of the total of cells positive for each marker to DAPI-positive cells in each bin (mean ± SEM). Analysis was performed using at least three independent experiments (three to four brains per genotype were quantified). The effect of the genotype on the distribution of cells within each bin was assessed using the Student test (*t*-*test*).

### Behavioral Testing

Behavioral experiments are briefly described below as they were performed as described previously (Moreau et al., 2010; Hilal et al., 2017; Ezan et al., 2021). All animal experiments were approved by the French Ministry of Research and local Ethics Committee and performed in accordance with the National Health and Medical Research Council Guidelines for the care and use of animals for scientific purposes. Male *Nex-Scrib* ^*−/−*^ cKOs littermate mice were 3 months old at the beginning of each study. Mice were individually housed 1 week before starting the behavioral experiment on a 12:12 h light–dark cycle and given ad libitum access to food and water. Behavioral studies were carried out during the light period. Behavior apparatuses were cleaned between mice with Phagospray.

#### Exploratory Activity and Anxiety-Like Behavior

In the elevated plus maze, the total distance travelled in all arms and the time spent in the open arms were measured over 5 min. In the open field test, the total distance travelled in the box and the time spent in the center are measured over 30 min. For the Y-maze, the total distance travelled, the number of arm entries, and the % of correct spontaneous alternance were measured. A correct alternation was defined as a non-repeated entry into the arms for three consecutive entries. Mice were recorded and tracked using Ethovision XT.

#### Locomotor Activity and Daily Rhythm

Animals were tracked in the actimetry cages (Immetronic) equipped with infrared sensors to detect locomotor activity. Mice activities were recorded 24 h per day for at least 3 days. The first 3 h of locomotor activity and the mean of the 2 last days were calculated.

#### Sensory–Motor Activity

For the rotarod test, mice were placed in an accelerated rotarod (4–40 rpm) over 5 min and the average latency to fall to the floor of the apparatus was measured for each testing day. Mice performed five trials over 3 days. The beam-walking assay was performed using a homemade experimental setup using a 1 cm square wood beam inclined at an angle of 10° from the ground. After 3 consecutive days of training, on the day of the test, we measured the number of paw faults and the mean time taken to traverse the beam into the enclosed box. During the inverted grid suspension test, mice were placed on a cage lid, which slowly turned, and the latency to fall was recorded in seconds over three trials of 5 min maximum.

### Statistical Analysis

Statistical analyses were carried out using the GraphPad Prism statistical package (GraphPad). Validation of normality of distribution and homogeneity of variance were performed, and unpaired Student’s two-tailed t-tests for two data sets were used to compare groups with similar variances. Details of n values are indicated along the *p* values in figure legends. *p* ≤ 0.05 was considered as statistically significant. Statistics was derived from at least three independent experiments and not from technical replicates. For behavior analyses, repeated measure ANOVA was used for the evaluation of the effect of genotype and time in actimetry and rotarod tests. The Bonferroni posthoc test was used when appropriate. Student’s t-test was used for comparing genotypes in other behavior tests.

## Data Availability

The raw data supporting the conclusion of this article will be made available by the authors, without undue reservation.
