# Supplementary figures and images for: Neuron-Specific Deletion of Scrib in Mice Leads to Neuroanatomical and Locomotor Deficits
Source: Front Genet. 2022 May 25;13:872700. doi: 10.3389/fgene.2022.872700 (PMC9174639; doi:10.3389/fgene.2022.872700)

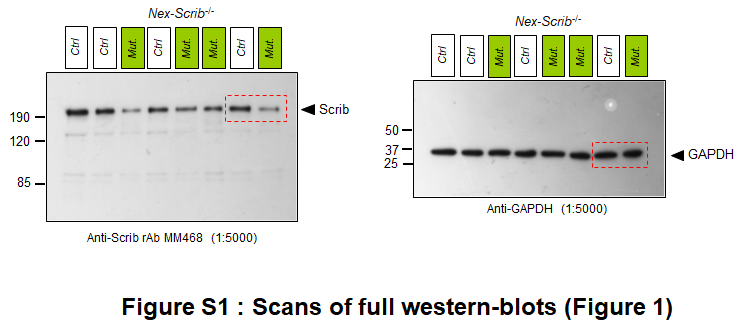

Supplement: Supplementary file 1 [file Image1.tif]
